# Comparative Analysis of Trigeminal Neuralgia Caused by Sole Arterial and Venous Compression: Clinical Features and Surgical Outcomes From 222 Cases

**DOI:** 10.3389/fneur.2021.634945

**Published:** 2021-04-28

**Authors:** Junwen Wang, Hongquan Niu, Kai Zhao, Kai Shu, Ting Lei

**Affiliations:** Department of Neurosurgery, Tongji Hospital, Tongji Medical College, Huazhong University of Science and Technology, Wuhan, China

**Keywords:** trigeminal neuralgia, microvascular decompression, posterior cranial fossa, neurovascular compression, outcome

## Abstract

**Background:** Compared with trigeminal neuralgia (TN) caused by arterial neurovascular conflict (NVC), the clinical characteristics and managements for TN with venous NVC are not well-established. This study aims to comparatively summarize the clinical features and surgical outcomes of microvascular decompression (MVD) for patients with TN caused by sole arterial and venous compression, with a particular focus on the morphological features of posterior cranial fossa (PCF).

**Methods:** A total of 222 patients with TN caused by sole arterial NVC (188/84.7%) and venous NVC (34/15.3%) underwent MVD in our department from January 2014 to December 2018. The patient data were analyzed retrospectively. Particularly, we focused on the potential impact of PCF on surgical outcomes.

**Results:** Compared with arterial NVC, V3 branch of the trigeminal nerve was more frequently involved in venous NVC (*p* = 0.009). The most common compression site was root entry zone for arterial NVC (68.6%) and midcisternal segment for venous NVC (76.5%) (*p* < 0.001). No serious post-operative complication was observed in the two groups. Both short- and long-term outcomes were relatively worse in venous NVC cases compared with arterial NVC cases (*p* = 0.001 and *p* = 0.030, respectively); and a dominantly higher rate of delayed cure was demonstrated in venous NVC cases (*p* < 0.001). TN patients with venous NVC revealed a more flat-shaped PCF than those with arterial NVC. Moreover, flat-shaped PCF morphometry was negatively correlated with surgical outcomes of TN patients with arterial NVC, but not with those of venous NVC cases.

**Conclusions:** MVD is an effective and safe treatment for patients with TN caused by either arterial or venous NVC. Patients with a more flat-shaped PCF might be vulnerable to venous compression. Our study demonstrated that PCF morphometry only affected the surgical outcomes of patients with TN caused by arterial NVC, but not the outcomes of those with venous NVC.

## Introduction

Trigeminal neuralgia (TN) is a common disorder characterized by paroxysmal, provokable, unilateral facial pain in the distribution of trigeminal nerve. The typical pain lasts only a few seconds to minutes but is described as violent and difficult to resist. According to the literature, the incidence of TN ranges approximately from 10 to 182 every 100,000 and gradually increases with age, especially in people over 60 years (1, 2).

The theory of neurovascular conflict (NVC) was first described by Dandy in 1934 and has been widely accepted as the etiology of TN (3). Accordingly, microvascular decompression (MVD) has been widely accepted as a preferred surgical treatment for medically intractable TN. Although TN is regularly caused by arterial compression of the nerve, venous compression or conflict can be another possible cause (1, 4–7). Numerous literatures explored the prognostic factors for outcomes of MVD for TN patients (2, 8, 9). However, in comparison with arterial NVC, the clinical characteristics and managements for venous NVC are not well-established. Most recently, a flat-shaped posterior cranial fossa (PCF) was identified as well as its association with the outcome of MVD for TN patients (9). However, the morphological features of PCF in different TN cases with arterial or venous NVC and its relationship with MVD outcomes have not been comparatively studied. In this retrospective study, we reviewed our experience in treating TN and explored the differences of clinical features and surgical outcomes between patients with TN caused by sole arterial and venous compression, with a particular focus on the PCF morphometry in line with the outcomes of TN patients.

## Materials and Methods

### Patients and Study Design

This study was approved by local ethical authorities of Tongji Hospital, Tongji Medical College, Huazhong University of Science and Technology, in accordance with the Helsinki Criteria. Written informed consent was obtained from each individual patient. From January 2014 to December 2018, 318 consecutive patients with TN underwent MVD in the neurosurgery department of Tongji Hospital, Wuhan, China. Of them, 222 classic TN patients with sole arterial or venous compression were enrolled in our present study. The exclusion criteria included inadequate radiological images, bilateral TN, previous microsurgical treatment, secondary TN, idiopathic TN, or even classic TN with simultaneous arterial and venous compression. The diagnosis was preoperatively conducted by two independent expert neurosurgeons through careful neurological examination. The type of NVC was identified according to the intraoperative findings. Patient data regarding clinical symptoms, neuroimaging characteristics, intraoperative findings, surgical outcomes, post-operative complications, and recurrence were reviewed retrospectively. The anatomic study of PCF morphometry was performed by using the methods described by Hamasaki et al.'s (10) and our previous publication (11). Briefly, the length (Y), width (X), and height (Z) of PCF were measured based on reconstructed coronal and sagittal T1-BRAVO images; and the volume of the PCF was calculated by using the formula $\frac{\pi }{6}$XYZ. Then, the ratio of X to Y (X/Y), X to Z (X/Z), and Y to Z (Y/Z) was also, respectively, investigated for further description of three-dimensional PCF features.

### Surgical Procedure

MVD was performed in all patients under neurophysiological monitoring. Generally, patients were placed in a “park bench” position and applied subjected suboccipital retrosigmoid craniotomy. After the dura was opened, cerebrospinal fluid (CSF) was drained sufficiently to relax the cerebellum so that the operation could be performed without the use of retractors. After the arachnoid membrane around the nerve was sufficiently dissected, the trigeminal nerve should be inspected circumferentially along its entire intracranial course from the root entry zone (REZ) at the brainstem laterally to Meckel cave. The NVC type, NVC site, NVC location, and NVC severity were identified and documented. After the offending vessel was detached from the nerve, Teflon sponges were gently inserted between the vessel and the nerve. For venous compression, the large trunk of vein, especially the superior petrosal vein (SPV) complex, should be retained. Only small veins and branches of super petrosal veins were coagulated and cut off to release the compression (5, 12, 13). In our present study, the offending vessels were divided into arterial and venous groups. The arterial NVC group was evaluated as the main trunk and branches of the vertebral artery (VA) and basilar artery (BA). The venous NVC group included SPV and its branches. The location of NVC was classified into three groups, including trigeminal REZ, midcisternal segment, and petrous segment. The site of NVC was evaluated as the site of offending vessels on the trigeminal circumference in a cross section according to previous publication (14, 15), in particular the conflict vessels that passed either medially or laterally to the superior border of the nerve leading to supero-medial or supero-lateral NVC, and the conflict vessels that compressed the inferior of the nerve leading to inferior NVC. In addition, the severity of NVC was evaluated according to Szapiro's classification (16): mild NVC, abuts against the nerve roots without obvious signs of compression; moderate NVC, nerve roots were obviously compressed with or without shift; and severe NVC, deformation or impression induced by compressed nerve root.

### Outcome Assessment

All patients were regularly followed up after hospitalization. Information was obtained from outpatient department visits and telephone interviews. For the phone interview, we offered a list of questions to assess pain level, side effects, and medication usage, which was described in previous publication (17). The pre- and post-operative evaluation was performed by the same group (consisted of two independent neurosurgeons) according to the same criteria to ensure the rigor of analysis. Patients were primarily evaluated at 1 week (short-term outcome) and at the last follow-up (long-term outcome) at least more than 1 year after surgery by using the Barrow Neurological Institute (BNI) pain intensity score. The outcome was classified as, namely, grade 1, no pain; grade 2, occasional pain, not requiring medication; grade 3, some pain controlled with medication; grade 4, some pain, not controlled with medication; and grade 5, severe pain or no pain relief. The curative effect was determined as success (BNI = 1) and failure (BNI ≥2). Consequently, recurrence was defined as any degree of pain repetition (BNI ≥2 on long-term outcome) after its disappearance (BNI = 1 in short-term outcome). Delayed cure was regarded as the residual facial pain after MVD (BNI ≥2 in short-term outcome) had obtained relief (BNI = 1 in long-term outcome). In addition, post-operative complications were documented.

### Statistical Analysis

Statistical analysis was performed by using IBM SPSS statistics 23.0 software. Normal distribution of continuous data was analyzed using Shapiro–Wilk test. All continuous data in our present study were not subject to normal distribution and were expressed as median and interquartile range (IQR). Continuous data were analyzed by the Mann–Whitney *U*-test (for non-parametric data). For categorized data, the two independent-samples tests were used to compare the differences. Statistical significance was established at *p* < 0.05.

## Results

The clinical information of all 222 TN patients in this study is summarized in Table 1. We included 75 men and 147 women with a median age of 57 years (range 50–80 years). The mean duration from symptom onset to surgery was 36 months (IQR 12–84 months). Pain triggers were identified in 178 cases (80.2%). The facial pain was located at the left side in 80 patients (36.0%) and at the right side in 142 patients (64.0%). Furthermore, the involved trigeminal nerve distributions were identified as V1 (5/2.3%), V2 (52/23.4%), V3 (79/35.6%), V1 + V2 (21/9.5%), and V2 + V3 (65/29.3%). Of the 222 patients, carbamazepine (CBZ) was preoperatively applied in 198 patients (89.2%), radiofrequency ablation in 16 patients (7.2%), and gasserian ganglion neurolysis in six cases (2.7%). And two patients (0.9%) did not receive any treatment before surgery. During operation, arterial NVC was observed in 188 cases (84.7%), while 34 cases (15.3%) were identified with sole venous NVC. The location of NVC was identified at REZ in 137 patients (61.7%), midcisternal segment in 69 patients (31.1%), and petrous segment in 16 patients (7.2%). Moreover, the site of NVC was recorded as supero-medial in 106 patients (47.8%), supero-lateral in 72 patients (32.4%), and inferior in 44 patients (19.8%). Additionally, mild NVC was evaluated in 35 patients (15.8%), moderate NVC in 138 patients (62.1%), and severe NVC in 49 patients (22.1%). As a result of short-term outcome evaluation, 175 patients (78.8%) were classified into success group (BNI score = 1) at 1-week post-operative evaluation, and 47 patients (21.2%) were evaluated as failure cases (BNI score ≥2) (Table 1). The mean follow-up duration was 45 months (IQR 32–92 months). For the long-term outcome evaluation, 180 patients (81.1%) were successful and 42 cases (18.9%) failed (Table 1). Eleven cases (5.0%) experienced recurrence, and 16 cases (7.2%) had a delayed cure.

**Table 1:** Clinical characteristics and outcomes of 222 classic TN patients.

**Variables**	**Number (%)**
Number of patients	*n* = 222
**Sex**	
Male/Female	75/147
Age (years)	57.00 (50.00, 65.25)
**Side**	
Left/Right	80/142
**Topography of facial pain**	
V1/V2/V3/V1+V2/V2+V3	5/52/79/21/65
**Type of pain**	
Type-1/Type-2	158/64
**Pain features**	
Pain duration (months)	36.00 (12.00, 84.00)
Trigger point	178 (80.2%)
**Treatment before MVD**	
CBZ application	198 (89.2%)
Radiofrequency ablation	16 (7.2%)
Gasserian ganglion neurolysis	6 (2.7%)
No previous treatment	2 (0.9%)
**Neurovascular identification on MRI**	
None	67 (30.2)
Compression	155 (69.68%)
**Type of NVC**	
Arterial/Venous	188/34
**Location of NVC**	
REZ/Midcisternal/Petrous	137/69/16
**Site of NVC**	
Supero-medial/Supero-lateral/inferior	106/72/44
**Severity of NVC**	
Mild/Moderate/Severe	35/138/49
Post-operative complications	32 (14.4%)
Follow-up duration (months)	45.00 (32.00, 92.00)
**Short-term outcome (BNI evaluation)**	
Success	175 (78.8%)
Failure	47 (21.2%)
**Long-term curative effect (BNI evaluation)**	
Success	180 (81.1%)
Failure	42 (18.9%)
Delayed cure	16 (7.2%)
Recurrence	11 (5.0%)

*BNI, Barrow Neurological Institute; CBZ, carbamazepine; MVD, microvascular decompression; NVC, neurovascular conflict; REZ, root entry zone; TN, trigeminal neuralgia*.

To explore the differences between TN patients with sole arterial and venous NVC, we comparatively analyzed their clinical data and summarized the findings in Table 2. We stated that there were no statistic differences regarding sex, age, pain duration, affected side, and post-operative complications between TN patients with arterial or venous NVC. Significant differences have been established with respect to the affected trigeminal nerve branches between the arterial and venous NVC groups. As a result, V3 branch was more frequently involved in venous NVC than in arterial NVC (85.3 vs. 61.2%) (*p* = 0.004). Compared with arterial NVC, the MRI scan revealed a significantly worse efficacy of NVC identification for venous NVC (*p* = 0.006). Moreover, the most common location of NVC was REZ for arterial NVC (68.6%) and midcisternal segment for venous compression (76.5%) (*p* < 0.001). Venous NVC usually occurred at the inferior and supero-lateral compartments (73.6%), while arterial NVC dominantly affected the supero-medial compartment of trigeminal nerve (51.6%) (*p* = 0.002). Mild NVC was more frequently identified in venous NVC (52.9%), while the definite moderate and severe NVC was more often detected in arterial NVC (91.0%) (*p* < 0.001). In addition, the values of X, X/Z, and Y/Z in the venous NVC group were remarkably larger, and the value of Z was significantly smaller than that in the arterial NVC group (X: *p* = 0.024; Z: *p* < 0.001; X/Z: *p* < 0.001; and Y/Z: *p* < 0.001), indicating that patients with a more flat-shaped PCF might be vulnerable to venous NVC. Moreover, the values of Y and X/Y showed no significant differences between these two groups. Subsequently, we performed a sex-dependent analysis (Table 3). As a result, we revealed that the volume of PCF and its substructure in female was smaller than that in male (X, Z, and volume: *p* < 0.001; Y: *p* = 0.002), which was in line with the previous publications (18). Moreover, a more flat-shaped PCF was identified in both female and male cases with venous NVC compared with arterial NVC. Additionally, post-operative evaluation demonstrated significantly better short- and long-term outcomes in the arterial NVC group compared with venous NVC, respectively (84.6 vs. 47.1% and 83.5 vs. 67.6%, *p* = 0.001 and *p* = 0.030, respectively). A dominantly higher rate of delayed cure was revealed in venous NVC cases than that in arterial NVC patients (23.5 vs. 4.3%, *p* < 0.001). Furthermore, there was no significant difference regarding post-operative recurrence between these two groups (5.3 vs. 2.9%, *p* = 0.999).

**Table 2:** Comparative analysis of classic TN patients with different type of NVC.

**Variables**	**NVC type**		***P*-value**
	**Arterial**	**Venous**	
Number of patients	*n* = 188	*n* = 34	
**Sex**			0.324
Male/Female	61/127	14/20	
Age (years)	59.00 (50.00, 66.00)	56.00 (51.30, 64.00)	0.311
Pain duration (months)	36.00 (12.00, 84.00)	36.00 (36.00, 112.00)	0.089
**Affected side**			0.384
Left/Right	70/118	10/24	
**Topography of facial pain**			0.004^**^
V1/V2/V3/V1+V2/V2+V3	4/51/58/18/57	1/1/21/3/8	
**Type of pain**			0.366
Type-1/Type-2	136/52	22/12	
**Neurovascular identification on MRI**			0.006^**^
None	50 (26.6%)	17 (50.0%)	
Compression	138 (73.4%)	17 (50.0%)	
**Location of NVC**			<0.001^***^
Root exit zone	129 (68.6%)	8 (23.5%)	
Midcisternal	43 (22.9%)	26 (76.5%)	
Petrous	16 (8.5%)	0 (0.0)	
**Site of NVC**			0.002^**^
Supero-medial	97 (51.6%)	9 (26.5%)	
Supero-lateral	61 (32.4%)	11 (32.4%)	
inferior	30 (16.0%)	14 (41.2%)	
**Severity of NVC**			<0.001^***^
Mild	17 (9.0%)	18 (52.9%)	
Moderate	124 (66.0%)	14 (41.2%)	
Severe	47 (25.0%)	2 (5.9%)	
Compliances	26 (13.8%)	6 (27.6%)	0.562
**PCF morphometry**			
Width (X, mm)	103.22 (100.48, 106.61)	107.72 (99.00, 114.50)	0.024^*^
Length (Y, mm)	71.97 (68.73, 73.50)	71.07(70.29, 73.42)	0.845
Height (Z, mm)	63.70 (61.22, 65.59)	59.62 (58.32, 61.88)	<0.001^***^
Volume (mm^3^)	245.35 (230.40, 260.90)	237.35 (220.83, 261.08)	0.238
Axial dimension (X/Y)	1,44 (1.37, 1.54)	1.48 (1.38, 1.60)	0.225
Coronal dimension (X/Z)	1.63 (1.55, 1.72)	1.76 (1.65, 1.91)	<0.001^***^
Sagittal dimension (Y/Z)	1.13 (1.07, 1.18)	1.19 (1.14, 1.24)	<0.001^***^
**Short-term outcome**			0.001^**^
Success	159 (84.6%)	16(47.1%)	
Failure	29 (15.4%)	18(52.9%)	
**Long-term outcome**			0.030^*^
Success	157 (83.5%)	23 (67.6%)	
Failure	31 (16.5%)	11 (32.3%)	
**Delayed cure**	8 (4.3%)	8 (23.5%)	<0.001^***^
Recurrence	10 (5.3%)	1 (2.9%)	0.999

* *P < 0.05*,

** *P < 0.01, and*

*** *P < 0.001*.

*BNI, Barrow Neurological Institute; NVC, neurovascular conflict; TN, trigeminal neuralgia; PCF, posterior cranial fossa*.

**Table 3:** Sex-dependent PCF morphometric differences in classic TN patients caused by sole arterial or venous NVC.

**Variables**	**Total patients (*****n*** **= 222)**		***P*-value**	**Male patients (*****n*** **= 75)**		***P*-value**	**Female patients (*****n*** **= 147)**		***P*-value**
	**Male**	**Female**		**Arterial**	**Venous**		**Arterial**	**Venous**	
Number of patients	*n* = 75	*n* = 147		*n* = 61	*n* = 14		*n* = 127	*n* = 20	
**PCF morphometry**									
Width (X, mm)	107.90 (103.30, 109.49)	101.73 (98.49, 105.39)	<0.001^***^	107.15 (102.70, 108.62)	110.23 (107.72, 116.25)	0.017^*^	101.67 (99.26, 104.52)	104.16 (96.48, 111.25)	0.540
Length (Y, mm)	72.15 (69.61, 74.42)	71.27 (67.99, 72.90)	0.002^**^	72.15 (69.43, 75.38)	72.71 (70.77, 73.60)	0.989	71.88 (67.24, 72.90)	70.58 (69.84, 71.95)	0.841
Height (Z, mm)	64.26 (62.26, 67.30)	62.50 (58.87, 64.48)	<0.001^***^	65.46 (62.75, 69.07)	61.09 (57.73, 65.22)	<0.001^***^	62.64 (59.43, 64.62)	58.89 (58.46, 60.76)	0.001^**^
Volume (mm^3^)	264.10 (247.00, 276.00)	237.50 (223.30, 251.30)	<0.001^***^	264.10 (247.70, 276.00)	262.35 (235.78, 274.23)	0.271	238.80 (223.50, 251.30)	230.10 (212.60, 250.98)	0.126
Axial dimension (X/Y)	1.49 (1.39, 1.58)	1.43 (1.37, 1.54)	0.147	1.45 (1.37, 1.57)	1.53 (1.45, 1.61)	0.131	1.43 (1.38, 1.54)	1.43 (1.36, 1.58)	0.906
Coronal dimension (X/Z)	1.64 (1.56, 1.75)	1.65 (1.56, 1.73)	0.989	1.60 (1.53, 1.71)	1.77 (1.66, 1.95)	<0.001^***^	1.64 (1.56, 1.72)	1.73 (1.62, 1.89)	0.005^**^
Sagittal dimension (Y/Z)	1.12 (1.08, 1.20)	1.14 (1.08, 1.20)	0.404	1.12 (1.07, 1.18)	1.20 (1.13, 1.26)	0.011^*^	1.14 (1.07, 1.18)	1.19 (1.16, 1.23)	0.001^**^

* *P < 0.05*,

** *P < 0.01, and*

*** *P < 0.101*.

*TN, trigeminal neuralgia; PCF, posterior cranial fossa*.

As shown in Tables 4, 5, the prognostic factors for both short- and long-term outcomes of TN patients with sole arterial and venous NVC were analyzed, respectively. In the arterial NVC group, the NVC site was not correlated with both short- and long-term outcomes. PCF morphological study revealed that flat-shaped PCF was a negative prognostic factor for both short- and long-term outcomes of TN patients with arterial NVC (Table 4). Nevertheless, no differences were established regarding topography of facial pain, site of NVC, and PCF morphometry for both short- and long-term outcomes of patients with venous NVC (Table 5).

**Table 4:** Outcomes after MVD for classic TN patients caused by sole arterial NVC.

**Variables**	**Short-term**		***P*-value**	**Long-term**		***P*-value**
	**Success**	**Failure**		**Success**	**Failure**	
Number of patients	*n* = 159	*n* = 29		*n* = 157	*n* = 31	
**Topography of facial pain**						
V1/V2/V3/V1+V2/V2+V3	4/44/52/11/48	0/8/6/6/9	0.134	4/46/50/11/46	0/5/8/7/11	0.047^*^
**Site of NVC**						
REZ/midcisternal/petrous	106/37/16	23/6/0	0.169	103/38/16	26/5/0	0.076
**PCF morphometry**						
Width (X, mm)	103.05 (99.58, 106.61)	104.52 (101.73, 106.35)	0.027^*^	103.30 (100.48, 106.73)	103.13 (104.54, 106.53)	0.922
Length (Y, mm)	72.05 (68.98, 73.50)	69.61 (65.76, 74.42)	0.252	71.73 (68.73, 73.26)	72.61 (65.76, 74.56)	0.061
Height (Z, mm)	63.79 (61.50, 65.59)	62.43 (60.10, 64.53)	0.137	63.85 (61.68, 66.05)	61.60 (58.30, 64.17)	0.002^**^
Volume (mm^3^)	244.40 (229.90, 264.40)	251.10 (231.90, 256.30)	0.950	245.90 (229.75, 263.15)	243.30 (231.90, 257.80)	0.486
Axial dimension (X/Y)	1.43 (1.36, 1.54)	1.52 (1.40, 1.57)	0.030^*^	1.44 (1.38, 1.55)	1.42 (1.33, 1.53)	0.225
Coronal dimension (X/Z)	1.61 (1.55, 1.70)	1.66 (1.57, 1.76)	0.031^*^	1.61 (1.54, 1.70)	1.66 (1.60, 1.75)	0.007^**^
Sagittal dimension (Y/Z)	1.13 (1.07, 1.18)	1.12 (1.03, 1.22)	0.931	1.12 (1.07, 1.17)	1.16 (1.11, 1.25)	0.003^**^

* *P < 0.05 and*

** *P < 0.01*.

*MVD, microvascular decompression; NVC, neurovascular conflict; TN, trigeminal neuralgia; PCF, posterior cranial fossa*.

**Table 5:** Outcomes after MVD for classic TN patients caused by sole venous NVC.

**Variables**	**Short-term**		***P*-value**	**Long-term**		***P*-value**
	**Success**	**Failure**		**Success**	**Failure**	
Number of patients	*n* = 16	*n* = 18		*n* = 23	*n* = 11	
**Topography of facial pain**						
V1/V2/V3/V1+V2/V2+V3	1/1/10/2/2	0/0/11/1/6	0.370	1/1/14/2/5	0/0/7/1/3	0.494
**Site of NVC**						
REZ/midcisternal/petrous	2/14/0	6/12/0	0.153	5/18/0	3/8/0	0.722
**PCF morphometry**						
Width (X, mm)	106.88 (99.73, 116.00)	108.09 (98.50, 112.50)	0.917	106.88 (101.92, 116.00)	108.45 (96.40, 112.00)	0.632
Length (Y, mm)	70.58 (69.84, 73.20)	72.12 (70.39, 73.51)	0.142	71.0 (70.36, 73.48)	72.04 (70.00, 73.30)	0.726
Height (Z, mm)	58.87 (58.46, 61.08)	60.45 (57.73, 63.68)	0.300	59.74 (58.46, 61.40)	59.20 (57.90, 65.22)	0.699
Volume (mm^3^)	230.55 (218.48, 257.03)	246.4 (223.00, 266.30)	0.351	238.60 (224.50, 258.90)	230.90 (214.10, 272.50)	0.868
Axial dimension (X/Y)	1.51 (1.39, 1.64)	1.47 (1.38, 1.59)	0.704	1.49 (1.39, 1.65)	1.47 (1.34, 1.59)	0.296
Coronal dimension (X/Z)	1.80 (1.68, 1.92)	1.73 (1.64, 1.93)	0.534	1.76 (1.66, 1.93)	1.70 (1.61, 1.90)	0.429
Sagittal dimension (Y/Z)	1.20 (1.18, 1.20)	1.18 (1.13, 1.25)	0.931	1.20 (1.16, 1.24)	1.18 (1.13, 1.24)	0.519

*MVD, microvascular decompression; NVC, neurovascular conflict; TN, trigeminal neuralgia; PCF, posterior cranial fossa*.

## Discussion

It is well-established that arterial compression of the nerve is the primary cause of TN (4). However, recent studies suggest that the involvement of veins is not a rare condition in TN (6, 7, 19–21). The chance of sole venous compression has been demonstrated to be around 10% (22). In our study, sole venous compression was found in 10.7% out of 318 cases, revealing a similar incidence to the previous reports. Compared with arterial NVC, venous NVC of TN is poorly understood regarding its clinical features and surgical outcomes. In the present study, we performed a comparative study between arterial and venous NVC and provided some new insights into vein-related issues in MVD surgery for TN.

Our study showed that the involvement of trigeminal nerve distributions in TN was different between cases with arterial and venous NVC. V3 branch was more frequently involved in venous NVC. According to the pathologic correlation of cross-section compartments of trigeminal nerve described by Sindou and Brinzeu (15), supero-medial compression of trigeminal nerve usually causes V1 pain and inferior compression causes V3 pain. However, V2 pain was not related to any types of compression. In the present study, we found that venous compression usually occurred at the inferior and supero-lateral compartments. This might be a possible explanation for the aforementioned differences of affected distributions between arterial and nervous compression. In addition, venous compression is generally weaker than arterial compression due to the lower vascular tension caused by relatively thin vessel wall and slow blood flow of veins. Therefore, mild NVC was more frequently identified in venous NVC, while the definite moderate and severe NVC was more often detected in arterial NVC.

The microsurgical management of offending arteries during MVD procedures has been well-studied in the past decades. However, the management of compressing veins is still controversial. Although the wall of vein is thinner than that of artery, venous compression tends to be a chronic and gradual pathologic process, which usually results in a stronger demyelination damage of the trigeminal nerve (8, 23). Therefore, MVD was still highly recommended for treatment of patients with TN caused by venous compression (6, 24). As known to all, dissection of nerve root and offending veins is more difficult and likely to cause laceration and hemorrhage (25). On the other hand, coagulation and division of the involved veins provide a more satisfactory and complete decompression, but they may increase local venous pressure causing cerebellum or brainstem infarction or hemorrhage, resulting in fatal complications (5, 7, 12, 13, 26). Therefore, some neurosurgeons suggested that small offending veins could be cut off directly without being divided (5, 6, 24). In our series, we would like to emphasize firstly that the SPV complex is covered by the circle of arachnoid membrane, so that a sufficient dissection of arachnoid membrane is the most important. Then, all compressed veins should be completely removed or decompressed, and only small intraneural veins could be sacrificed. Our data showed that no post-operative complications (cerebellum or brainstem infarction or hemorrhage) were observed in cases with vein division. Literatures (24, 27) and our data demonstrated that MVD for TNs caused by arterial NVC displayed better surgical effects in both short-term and long-term evaluations. However, a delayed cure more dominantly occurred in venous NVC cases in comparison with the arterial NVC group, which suggested that it should take a longer period to monitor pain relief in these patients after MVD surgery.

During the past decades, the PCF morphometry has been widely explored in TN. Previous studies indicated that the PCF morphometry might be similar in healthy subjects and classic TN patients (18, 28, 29). In addition, a smaller PCF was merely characterized as TNs without NVC, so-called idiopathic TNs (18). However, there were differences regarding the study design between previous works and our present work. On the one hand, we focused on the comparison of classic TN caused by sole arterial and venous NVC, which were not described in the previous reports. On the other hand, we also focused on the evaluation of PCF on the surgical outcomes of TNs, which was also absent in the previous reports. Most recently, Fukuoka et al. found that a more flat PCF might be associated with poor outcome of MVD for TNs (9). As reported by literatures, other anatomic features were also correlated with outcomes of MVD for TNs (30–32). Therefore, PCF morphometric features might influence the outcome of MVD. In our study, we focused on the impact of PCF morphometry in TNs with pure arterial and venous NVC. As a result, the volume of PCF was measured similarly in both arterial and venous compression TNs, but a more flat-shaped (short along the superior–inferior axis and longer along the left–right axis) PCF was identified in the venous NVC group in comparison with the arterial NVC group, indicating that the PCF morphometry might influence the vascular development and in turn result in a different NVC in the prevalence of TNs. Of the 188 TNs caused by arterial compression, PCF morphometry was positively associated with MVD outcomes, which was in accordance with the previous reports (9). Although TNs with venous compression displayed a more flat-shaped PCF, the outcomes of MVD for these patients were not influenced by PCF morphometry. Maybe an evaluation of a larger series including TN and healthy cases in the future would help to reach a more accurate understanding of the morphometric changes in this disease. Indeed, a flat PCF would result in a smaller space for surgical procedures. However, experienced neurosurgeons and modern surgical techniques, such as high-definition microscopy and endoscopy, could avoid the risk of increased difficulties in treating these patients. As a result, we would not consider a flat PCF as a factor enough to influence the methodological surgical issues. We would believe that flat PCF exerts a more important role in pathological changes of nerve demyelination followed by NVC.

In this study, we demonstrated several differences of clinical features, surgical outcomes, and PCF morphometry between TN patients with sole arterial and venous NVC, identified prognostic factors for MVD outcomes of TNs, and particularly provided some new insights for MVD performance in TN with venous NVC. However, limitations could not be avoided in this study. The number of patients involved in this study was relatively small, particularly for patients with venous compression. No healthy or control cases were included in this work, which might lead to bias caused by case enrollment. Therefore, further studies should be continuously performed in large patient populations.

## Conclusion

The involvement of venous compression should be taken into account for TN patients with obvious facial pain in V3 distribution of trigeminal nerve. MVD is an effective and safe treatment strategy for patients with TN caused by either arterial or venous compression. Particularly for TN patients with venous NVC, only small intraneural veins could be sacrificed, while the other veins should be rigorously preserved and decompressed during surgical procedure. In addition, TN patients with venous NVC revealed a more flat-shaped PCF than those with arterial NVC. However, PCF morphometry is only negatively correlated with MVD outcomes of patients with TN caused by arterial NVC, but not with those by venous NVC.

## Data Availability Statement

The original contributions presented in the study are included in the article/supplementary material, further inquiries can be directed to the corresponding author/s.

## Ethics Statement

The studies involving human participants were reviewed and approved by Tongji Hospital, Tongji Medical College, Huazhong University of Science and Technology. The patients/participants provided their written informed consent to participate in this study.

## Author Contributions

KS and TL designed and conducted this project. KZ, JW, and HN collected the data and run statistical analysis. KZ and JW wrote the original manuscript. All authors revised the manuscript and approved the submission.

## Conflict of Interest

The authors declare that the research was conducted in the absence of any commercial or financial relationships that could be construed as a potential conflict of interest.

## Abbreviations

BA: basilar artery
BNI: Barrow Neurological Institute
CPA: cerebellopontine angle
CSF: cerebrospinal fluid
IQR: interquartile range
MRI: magnetic resonance imaging
MVD: microvascular depression
NVC: arterial neurovascular compression
PCF: posterior cranial fossa
REZ: root entry zone
SPV: superior petrosal vein
TN: trigeminal neuralgia
VA: vertebral artery.
